# Synthesis, Characterization, and Biosorption of Cu^2+^ and Pb^2+^ Ions from an Aqueous Solution Using Biochar Derived from Orange Peels

**DOI:** 10.3390/molecules28207050

**Published:** 2023-10-12

**Authors:** Felicia Omolara Afolabi, Paul Musonge

**Affiliations:** 1 Department of Chemical Engineering, Durban University of Technology, Durban 4001, South Africa; 2 Institute of Systems Science, Durban University of Technology, Durban 4001, South Africa; paulm@dut.ac.za; 3 Faculty of Engineering, Mangosuthu University of Technology, Durban 4031, South Africa

**Keywords:** biochar, kinetics, equilibrium, adsorption, binary systems

## Abstract

In this study, orange peel (OP) biochar was used as a bio-sorbent for the removal of copper and lead from wastewater in single and binary systems. The equilibrium and kinetic studies were conducted at a pH value of 5, which was the maximum adsorption pH value for both metal ions. The equilibrium studies were investigated at a varying initial concentration (10–200 mg/L) with a constant dosage of 0.1 g, while the kinetic studies were conducted at a fixed initial concentration of 200 mg/L with a constant dosage of 1 g/L for both single and binary systems. The maximum adsorption capacity of the orange peel biochar was 28.06 mg/g, 26.83 mg/g, 30.12 mg/g and 27.71 mg/g for single Cu^2+^, binary Cu^2+^, single Pb^2+^ and binary Pb^2+^ systems, respectively. The Langmuir isotherm model fitted the experimental data, suggesting that adsorption occurred on a monolayer, while the pseudo-second-order model performed well with the kinetic data. The point of zero charge (pH_pzc_) of the orange peel biochar was found to be 10.03, which revealed that the surface of the bio-sorbent contains basic groups. A Fourier infrared transform (FTIR) spectroscope and scanning electron microscope, coupled with energy dispersive x-ray (SEM-EDX) and x-ray diffraction analyses, were used to determine the functional groups, surface morphology, and inorganic elements present on the surface of the bio-sorbent, respectively. The results obtained have shown that orange peel biochar is efficient for the removal of Cu^2+^ and Pb^2+^ ions from an aqueous solution.

## 1. Introduction

Increasing urbanization and industrialization have led to surged industrial and sewage effluents in most developing countries. Serious environmental problems are generated from the improper disposal of these effluents into water streams and soils. The effluents contain heavy metals which are toxic and non-biodegradable. These heavy metals contaminate water bodies, thereby causing a severe impact on human health [1]. Therefore, stringent rules and regulations are put in place by governments to guide the disposal of such wastes.

Copper and lead are the most pervasive heavy metals found in water because these metals are used as raw materials for many industrial activities, such as explosive manufacturing, pigmenting, photography, smelting, fuelling, printing, and storing batteries [2,3]. The presence of copper and lead in water or food can cause danger to human health, and contribute to liver, kidney, and cardiovascular diseases, Wilson disease, insomnia, seizures, intellectual disability, and damage to the nervous system [4,5]. Hence, these toxic contaminants must be removed from drinking water, streams, groundwater, and wastewater, to safeguard human lives and the environment. Several treatment techniques have been used successfully to remove heavy metals from aqueous solutions, such as chemical precipitation, ion exchange, electrochemical reduction, membrane filtration, and adsorption. However, these methods have their drawbacks, which include the high cost of chemicals, incomplete removal of pollutants, high energy usage, and the generation of a large volume of sludge, which in turn need to be disposed of [4]. 

Oranges are one of the fruits largely consumed in many parts of the world and thus contribute significantly to the amount of waste that is generated in households, marketplaces, and fruit processing industries. Approximately 45 million tons of oranges are produced in the world per year, while 20 million tons of orange peels are generated from food industries [6]. These fruit peels are mostly disposed of through landfilling, composting, and open-air burning. These disposal techniques pose serious environmental pollution issues, which include the generation of greenhouse gases through landfilling and toxic compounds produced from open-air burning, while composting could result in bad odours. Orange peels consist of cellulose, hemicellulose, lignin, and pectin, which comprise functional groups that can enhance the adsorption of heavy metals. 

Recently, the use of low-cost bio-sorbents in the remediation of wastewater has been an area of focus for researchers because they are readily available, sustainable, efficient, and economical. Several bio-sorbents have been used for the removal of heavy metals from wastewater, such as banana peels, watermelon rind, potato peel, mango peel, rice husk, sugarcane bagasse, etc. [7,8,9]. The chemical and physical characteristics of these sorbent materials, such as their morphological features, the pH value at the point of zero charge, functional groups, elemental composition, and crystallographic characteristics, are significant for assessing their potentiality and efficiency for removing contaminants from wastewater [10,11,12]. The biosorption process depends on the metal and the properties of the biomass surface. Many researchers have reported the physical and chemical properties of bio-sorbents and how it affects the adsorption mechanism. Orange peel biochar was characterized using SEM, EDX, and FTIR analyses before and after the adsorption of Cu^2+^ and Cd^2+^ in previous studies conducted by the authors of [13]. The authors confirmed that the successful adsorption of the metal ions was due to the strong physiochemical interactions between the biochar and the metal ions. 

Pyrolysis is the thermal decomposition of biomass material in the absence of air to produce bio-oil, gases, and biochar. The pyrolysis process can be optimized by changing the operating parameters of the process, such as the temperature and heating time. Pyrolysis of biomass at high temperatures (700 °C and above) with a short heating time will produce bio-oil and gases, while pyrolysis at low temperatures (500 °C) with a longer heating time will yield biochar [6]. However, biochar produced by pyrolysis at 500–700 °C contains more micropores and a larger surface area [14]. Biochar produced from fruit peels has been used for adsorption studies [12,15]; however, the binary adsorption of heavy metals from aqueous solutions using orange peel biochar has not been discussed. Yang et al. evaluated the adsorption capacity of Enteromorpha-derived biochar to adsorb heavy metals from seawater. The adsorption capacity of Cu^2+^ and Pb^2+^ was favoured by high pH conditions, while elevated salinity had a relatively weak negative effect on the adsorption process [16]. Katiyar et al. studied the adsorption of Cu^2+^ on seaweed (*Ascophyllum nodosum*)-derived biochar at 700 °C [17]. The highest removal efficiency of Cu^2+^ from the aqueous solution was >99%, with a 223 mg/g adsorption capacity at a pH value of 5. This study is based on the removal of copper and lead from an aqueous solution using biochar. It is rare to find a single heavy metal in wastewater; therefore, the study of the simultaneous removal of heavy metals from wastewater is expedient. 

## 2. Results and Discussion

### 2.1. Point of Zero Charge of Orange Peels (pHpzc)

**Figure 1:**
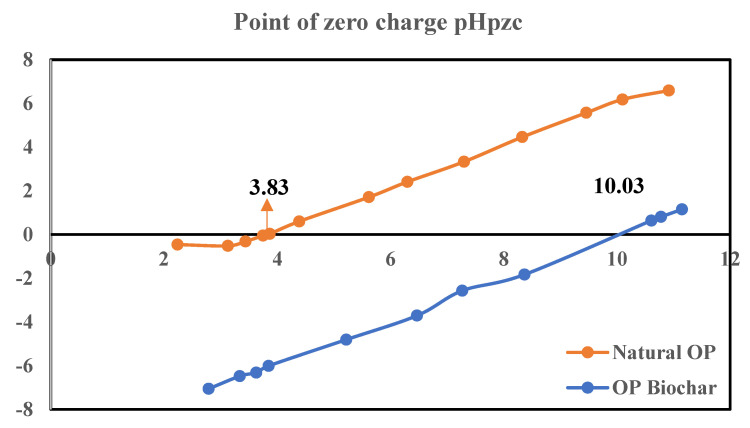
Point of zero charge (pHpzc) of natural OP and OP biochar.

The point of zero charge of an adsorbent is the point at which its charge is zero. From Figure 1, the pHpzc of natural OP and OP biochar is 3.83 and 10.03, respectively. The pyrolysis process changed the surface of the natural OP from acidic to basic, which suggests that the functional groups consist of basic groups. The increase in the surface pH value of the biochar due to the pyrolysis temperature can be explained by the volatilization and decomposition of organic acids and phenolic substances during the pyrolysis process [18].

### 2.2. Characterization of Bio-Sorbent

#### 2.2.1. FTIR Spectroscopy Analysis

**Figure 2:**
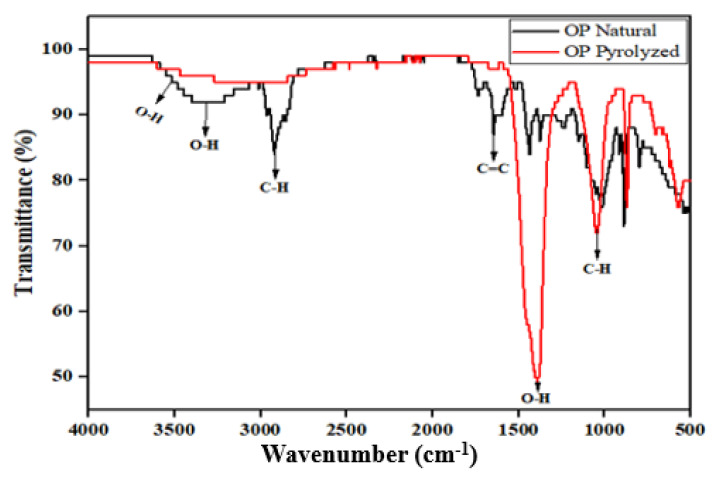
FTIR analysis of natural OP and OP biochar.

The FTIR analysis is very important for determining the functional groups that are present on the surface of an adsorbent. The FTIR analysis is represented with the plot of percentage transmittance against wavenumber. The sharp peaks are significant and help to identify various functional groups with their stretching bands. In Figure 2, the natural OP consists of peaks such as 3330 and 1316.4 cm^−1^, which are denoted by the O-H group, alcoholic stretching. The peaks at 2920.41 and 1015.77 cm^−1^ correspond to the C-H group, alkane sharp stretching band. The peak at 1733 cm^−1^ signifies the C=C, alkene group. There are significant changes in the absorption peaks after the pyrolysis process of OP. The broad O-H band at 3330 cm^−1^ with 89.04% transmittance has shifted and become flat. In addition, the C-H and C=C groups at wavenumber 2920.41 and 1733 cm^−1^, respectively, disappeared. However, significant sharp peaks are noticed at 1316.4 and 1015.77 cm^−1^, which correspond to O-H and C-H stretching vibrations. These peaks are well pronounced, which suggests that certain minerals consisting of carbon, oxygen, and hydrogen are present on the surface of the biochar.

#### 2.2.2. X-ray Diffraction 

**Figure 3:**
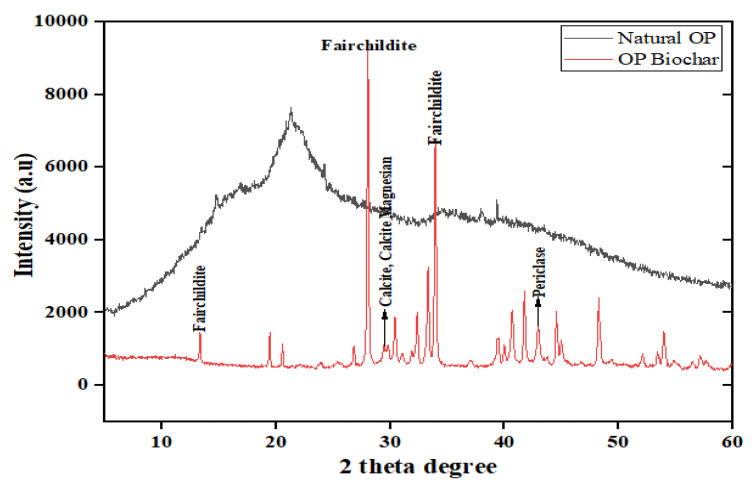
X-ray diffraction of natural and biochar OP.

The crystal structures of natural OP and OP biochar were determined using a multipurpose X-ray diffractometer D8-Advance from Bruker AXS (Oxford X-Max detector, Oxford, UK and INCA software v7.3, Stuttgart, Germany). It was operated in a continuous ϑ–ϑ scan in locked coupled mode with Cu-K_α_ radiation. The measurements were carried out in a range of 2θ with a step size of 0.034°, as shown in Figure 3. The XRD of natural OP revealed that the peaks were not excessively sharp, which suggests that the biomass is amorphous. However, the biochar profile showed various sharp peaks, which means that there were significant changes in the structure of the biomass after pyrolysis at 500 °C. The notable peaks are fairchildite, (K_2_Ca(CO_3_)_2_) visible at 13.5°, 28°, 33.5°, and 34°, calcite (CaCO_3_) and calcite magnesian ((Ca, Mg)CO_3_) at 29.5°, and periclase (MgO) at 43°. After pyrolysis, there was loss of amorphous phase, which may be due to the cracking of bonds and degradation of acidic functional groups present in the raw materials, and possibly increasing the ash content. 

#### 2.2.3. Scanning Electron Microscope (SEM) Analysis

**Figure 4:**
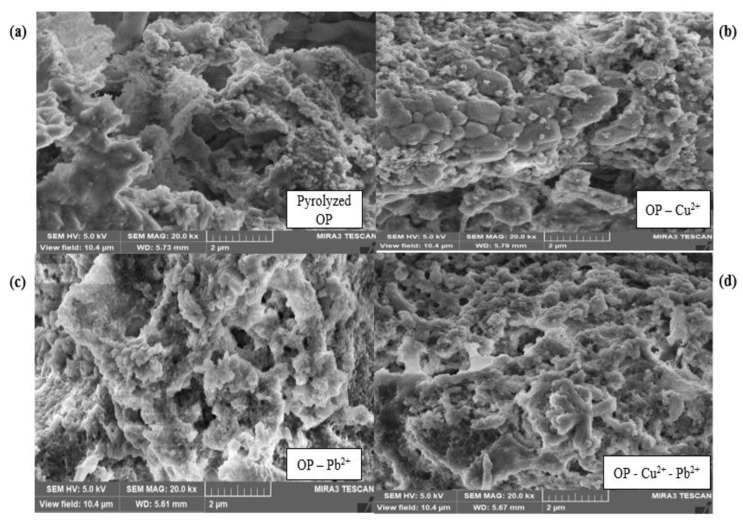
SEM analysis of orange peel biochar before and after adsorption (**a**) pyrolyzed OP (**b**) OP loaded with Cu^2+^ (**c**) OP loaded with Pb^2+^ (**d**) OP loaded with Cu^2+^ and Pb^2+^.

The SEM analysis explains the morphological structure of the bio-sorbent before and after the adsorption of Cu^2+^ and Pb^2+^, as shown in Figure 4. Figure 4a showed the surface structure of the OP biochar before the sorption process. The surface structure of the OP biochar before adsorption showed a porous, irregular, and heterogenous surface. The porous and irregular surface structure of the biochar showed the potential of the biochar to adsorb the desired metal ions, since the pores provide adequate adsorption sites. The surface structure of the OP biochar loaded with Cu^2+^ showed the presence of white particles, which is due to the sorption of Cu^2+^ ions on the surfaces and the pores of the bio-sorbent (Figure 4b). Figure 4c revealed that the surface of the bio-sorbent after the adsorption of Pb^2+^ became shiny. The surface of the bio-sorbent became shiny and whitish after the adsorption of Cu^2+^ and Pb^2+^ ions (Figure 4d). Therefore, the presence of Cu^2+^ and Pb^2+^ ions on the surface of the biochar after adsorption suggested that physiochemical interactions occurred between the functional groups on the surface of the bio-sorbent and the metal ions in the solution. These results were further confirmed by the elemental composition analysis obtained from the EDX analysis.

#### 2.2.4. Energy Dispersive X-ray

**Figure 5:**
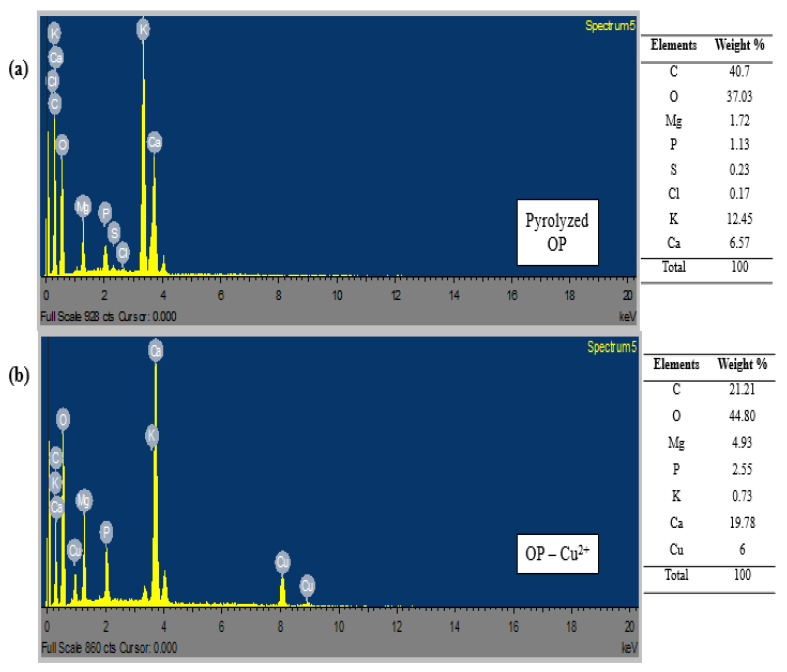

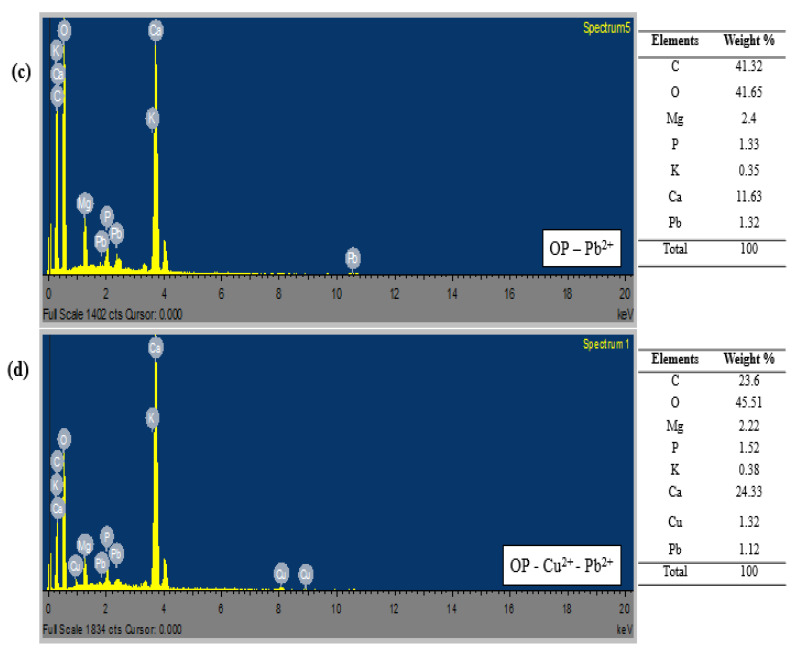
EDX analysis of orange peel biochar before and after adsorption: (**a**) pyrolyzed OP, (**b**) OP loaded with Cu^2+^, (**c**) OP loaded with Pb^2+^, and (**d**) OP loaded with Cu^2+^ and Pb^2+^.

An energy dispersive x-ray was used to determine the inorganic elements present in the biochar sample of orange peels before and after adsorption studies. The elemental compositions are represented in the tables indicating the respective weight percentage of each element. The elemental composition of OP biochar before adsorption showed that the bio-sorbent possesses a high percentage of carbon (C, 40.7%), followed by oxygen (O, 37.03%), as depicted in Figure 5a. The presence of other elements, such as potassium (K, 12.45%), calcium (Ca, 6.57%), magnesium (Mg, 1.72%), phosphorus (P, 1.13%), silicon (Si, 0.23%), and chlorine (Cl, 0.17%), shows that OP biochar has the potential for heavy metal adsorption [13]. However, the elemental composition in the post-sorption samples revealed significant changes in the weight percent of the elements (Figure 5b–d). The EDX results after adsorption showed the presence of carbon, oxygen, magnesium, phosphorus, potassium, calcium, and the adsorbed metal ions. Hence, these EDX results confirmed that Cu^2+^ and Pb^2+^ were successfully adsorbed in a single and binary solution on the surface of the OP biochar, which is due to the strong physiochemical interactions between the metal ions in the solution and the elements on the surface of the bio-sorbent. 

### 2.3. Effect of pH Value

**Figure 6:**
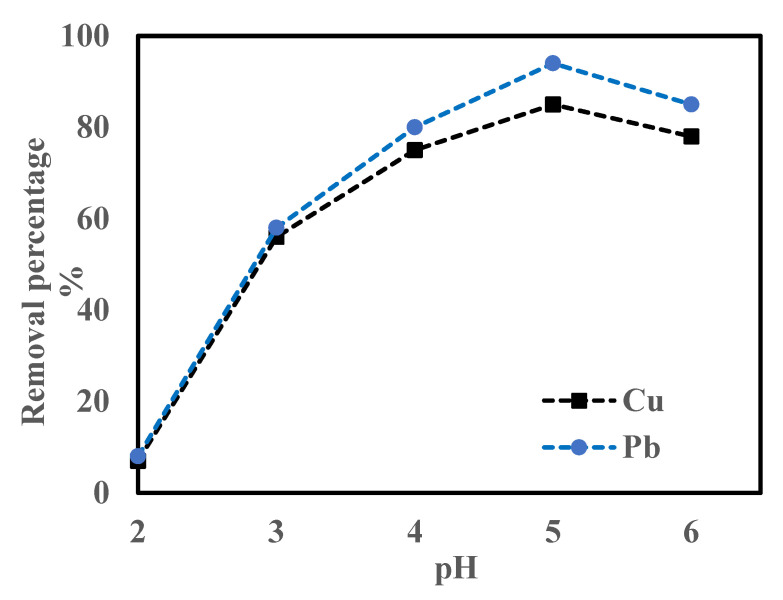
Effect of pH value on the adsorption capacity of Cu^2+^ and Pb^2+^.

The adsorption process is greatly affected by the pH value of the solution [19]. The pH value of the aqueous solution and the point of zero charge of the bio-sorbent play an important role in determining the mechanism of the adsorption process. As the pH value of the solution changes, the adsorption efficiency also changes due to the mobility of the hydroxyl (OH^−^) and hydrogen (H^+^) ions in the solution. Batch tests were performed in the pH range of 2 to 6 to investigate the influence of the pH value, as depicted in Figure 6. A total of 1.0 g of orange peel biochar was agitated at a contact time of 1 h using 100 mg/L of Cu^2+^ and Pb^2+^, respectively. 

The initial pH value of the solution is a critical parameter in the metal adsorption process, which is obvious in the results obtained in this study, where the percentage removal of the metal ions increased with an increase in the pH value of the solution, until a maximum metal uptake was reached at a pH value of 5.0. The removal efficiency of the metal ions increased from 7% to 85% and 8% to 94% for Cu^2+^ and Pb^2+^, respectively, as the pH value of the solution increased from 2 to 6 (Figure 6). A very low percentage removal of both metal ions was observed at the strong acidic region (pH value of 2), which suggests that an interaction occurred between the metal ions present on the sorption sites of the biochar and the excess hydrogen in the aqueous solution. At a low pH value, excess hydronium ions compete with the heavy metal ions in the solution to access the active sites of the bio-sorbent, thus resulting in the reduction of heavy metal adsorption uptake. At a pH value of 5, a decreased positive surface charge, together with the presence of more negative charges, resulted in a higher percentage of removal. However, the precipitation of hydroxides of the metals at a pH value of 6 lowered the percentage removal of the metal ions, thus implying that a pH value of 5 was appropriate for the adsorption of Cu^2+^ and Pb^2+^ in this study. 

### 2.4. Adsorption Isotherm

**Figure 7:**
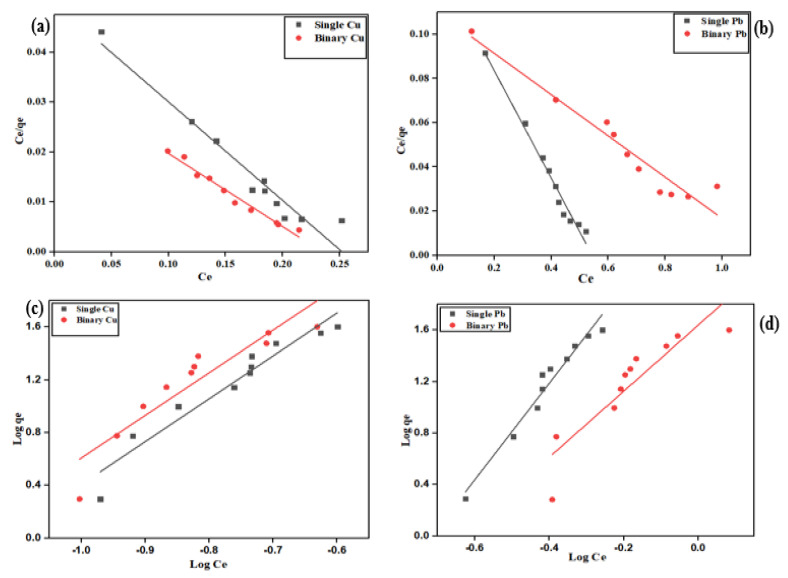
Adsorption isotherm of Cu^2+^ and Pb^2+^ onto orange peel biochar in the single and binary system at a pH value of 5. The symbols are the experimental data while the solid lines are the linear fittings of (**a**) Cu^2+^ Langmuir adsorption, (**b**) Pb^2+^ Langmuir adsorption, (**c**) Cu^2+^ Freundlich adsorption, and (**d**) Pb^2+^ Freundlich adsorption.

The adsorption isotherm studies were conducted with varying initial metal concentrations (10–200 mg/L) and a constant adsorbent dosage (0.1 g). The adsorption studies were carried out in single and binary systems with equal concentrations. The experimental data and the linear fit of the isotherm models used for the bio-sorption of Cu^2+^ and Pb^2+^ in both systems are depicted in Figure 7. Table 1 shows the isotherm model parameters for the single and binary bio-sorption systems. 

The maximum adsorption capacity of orange peel biochar was 28.06 mg/g, 26.83 mg/g, 30.12 mg/g, and 27.71 mg/g for single Cu^2+^, binary Cu^2+^, single Pb^2+^, and binary Pb^2+^ systems, respectively. The adsorption capacity of the biochar increased with an increase in the initial metal concentration. The adsorption uptakes of both metals were lower in the binary system than in the single system, which shows the effect of the co-existence of metal ions in a solution. In both single and binary systems, Pb^2+^ was more highly adsorbed than Cu^2+^, which suggests that orange peel biochar had a greater affinity for Pb^2+^. 

The Langmuir isotherm model has a basic property expressed as the dimensionless constant (R_L_), also known as the separation factor. R_L_ is a value used to predict whether the adsorption isotherm is favorable (0 < R_L_ < 1), unfavorable (R_L_ > 1), linear (R_L_ = 1), or irreversible (R_L_ = 0). In this study, the separation factor R_L_ was within the range of 0.49 to 0.82, which indicates that the adsorption process is favorable, since the value is between 0 and 1. 

It was observed that higher values of R^2^ were obtained for the Langmuir isotherm model in the single and binary systems of Cu^2+^ and Pb^2+^ as compared to Freundlich isotherm model. This suggests that the equilibrium data obtained for the adsorption of Cu^2+^ and Pb^2+^ are in better agreement with the Langmuir isotherm model. These results are in agreement with results reported in previous studies [20]. In addition, this indicates that the surface of the biochar is regular and heterogenous, which is consistent with the results of the SEM and EDX analyses obtained before and after adsorption of Cu^2+^ and Pb^2+^. In the Freundlich isotherm model, the coefficient ‘n’ relates to the intensity, heterogeneity, and type of the adsorption process. When n = 1, the adsorption is linear; when n < 1, the adsorption is a chemical process; and when n > 1, this means the adsorption is a physical process. In this study, the value of n was in the range of n < 1, which suggests that the adsorption process is chemical. This can further be explained by the EDX results after adsorption, which showed the replacement of elements present on the surface of the biochar with Cu^2+^ and Pb^2+^ after adsorption. 

### 2.5. Adsorption Kinetic

**Figure 8:**
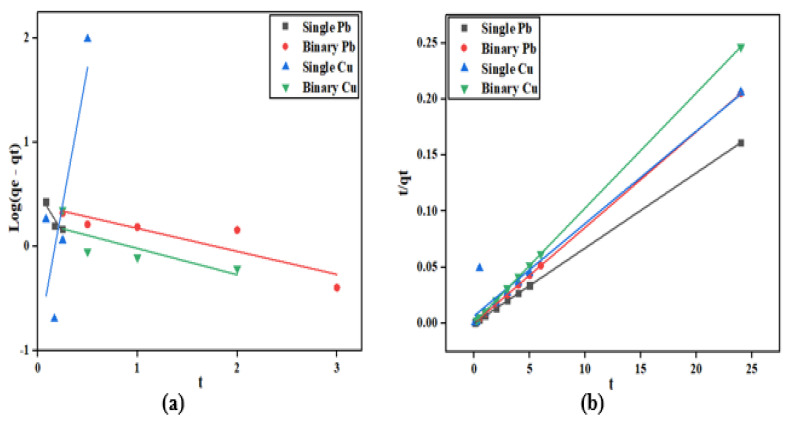
Kinetic plot of Cu^2+^ and Pb^2+^ at different initial concentrations in the single and binary system: (**a**) Pseudo-first order single and binary system and (**b**) Pseudo-second order single and binary system.

The adsorption kinetics were explained by fitting the experimental data with the linearized form of the pseudo-first order and pseudo-second order kinetic model, as depicted in Figure 8, while the model parameters are presented in Table 2. The kinetic studies were conducted at a fixed initial concentration of 200 mg/L for single and binary systems of Cu^2+^ and Pb^2+^, to explain the effect of the co-adsorption or co-existence of the metal ions. The experimental kinetic data of Cu^2+^ and Pb^2+^ in both systems at the initial concentration performed well with the pseudo-second-order model, which gave a correlation coefficient (R^2^) equal to 1. This R^2^ value suggests that the adsorption process is chemically controlled. The equilibrium adsorption capacity of Cu^2+^ and Pb^2+^ increased with the increasing initial concentration for both single and binary systems. In addition, the value of K_2_ for Pb^2+^ in the binary system was higher than Cu^2+^ which suggests that the co-existence of Cu^2+^ ions in the solution did not affect the adsorption rate of Pb^2+^. This implies that orange peel biochar has a higher affinity for Pb^2+^ than Cu^2+^. 

### 2.6. Competitive Adsorption Analysis

Wastewater is a complex system comprising different types of heavy metals and interfering ions which require a good, functionalized adsorbent for efficient removal in a competitive environment. Hence, the adsorption performance of Cu^2+^ and Pb^2+^ in a binary system using biochar derived from orange peels was investigated. The adsorption capacity of Cu^2+^ and Pb^2+^ increased with an increasing initial concentration of each metal ion because of improved transport dynamics between the heavy metal ions and the adsorbent. The adsorption behaviour of orange peel biochar for the removal of Cu^2+^ and Pb^2+^ followed the Langmuir isotherm model, suggesting a monolayer sorption process. For the Cu^2+^ and Pb^2+^ binary system, the adsorption performance of the two metal ions showed little changes as compared with the adsorption capacities in the single system due to the competitive behaviour. 

Generally, the competitiveness of Pb^2+^ and Cu^2+^ ions for the active sites of the adsorbent material can be explained using the parameters characterizing the binding strength of metal ions, such as the atomic mass, ionic radius, electronegativity, hydration radius, covalent binding, and hydrolysis constant, as shown in Table 3. The electrostatic binding of metal ions is related to a change in the inclination of the water molecules that have been hydrated. There may be a change in state, and the metal ion may accumulate at the interface of the bio-sorbent if the hydrated water molecules are not tightly bound by the metal ion. When comparing the crystal radii of ions with the same charge, such as Pb^2+^ and Cu^2+^, it is typical to find that larger, weaker hydrated ions are primarily deposited at the interface [21]. In essence, Pb^2+^ has a lower hydration ion radius than Cu^2+^ despite having a larger propensity for adsorption on the bio-sorbent. This explains why ions with a lower degree of hydration tend to collect at the interface. Additionally, the inclination and ability for the metal ion to establish covalent bonds with the functional groups on the surface of the bio-sorbent increase with the covalent binding index [22]. Larger ions can therefore fit into a binding site and bind to multiple groups on an adsorbent surface at once. This explains why Pb^2+^ has a stronger affinity.

### 2.7. Possible Adsorption Mechanism

**Figure 9:**
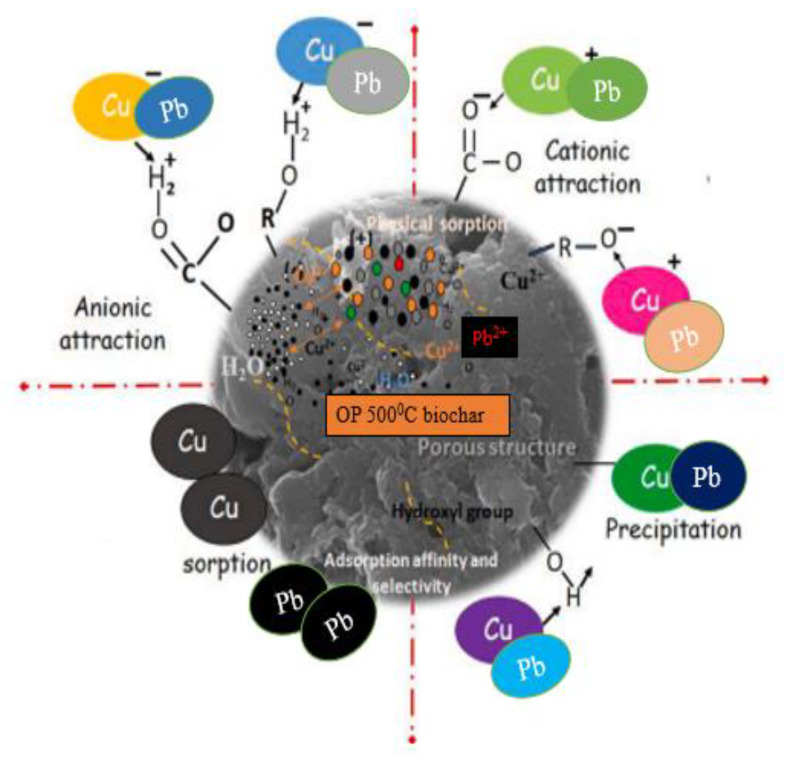
Adsorption mechanism of Cu^2+^ and Pb^2+^ onto orange peel-derived biochar.

Orange peel biochar consists of a rich chemical composition; some of the chemical groups present on the surface of the bio-sorbent can participate in the adsorption reaction. The complexity of the chemical composition of the orange peel biochar can result in a combination of multiple mechanisms in the adsorption process. During pyrolysis, the carbonization process results in the selective breakdown of elements into gases and volatile compounds. One of the reasons to improve the polarity of biochar, increase the pore volume and surface area, and enhance the developed void structure in the produced biochar, is that it helps the O/C and H/C ratios in the resulting biochar that determine the features and type of interactions of the biochar based on the reactive groups. The adsorption mechanism of Cu^2+^ and Pb^2+^ onto orange peel-derived biochar can be linked to numerous interactions between the adsorbent and the adsorbate in the aqueous solution. The adsorption mechanism heavily involved electrostatic attractions, surface precipitation, and chemical sorption according to the findings of the material characterization and adsorption isotherm studies (Figure 9). 

Considering the results of the FTIR analysis and the changes in the pH values of the biochar used in this study, there are interactions between the functional groups present on the surface of the biochar and the metal ions (Cu^2+^ and Pb^2+^). In addition, the EDX results after adsorption showed that some elements disappeared and were replaced with metal ions in the solution. In the aqueous solution, copper and lead hydroxo complexes may be formed at intermediate pH levels, which can result in the precipitation in the form of hydroxides of the metal ions on the surface of the biochar [20]. The results of the FTIR analysis of the biochar showed a pronounced peak for hydroxyl, carboxyl, and carbonyl functional groups, which can create electrostatic attraction with the metal ions. 

### 2.8. Comparison of Pb^2+^ and Cu^2+^ Uptake Using Orange Peels Biochar and Other Bio-Sorbents

The adsorption capacity of Cu^2+^ and Pb^2+^ onto orange peel biochar in single and binary systems was 28.06 mg/g, 26.83 mg/g, 30.12 mg/g, and 27.71 mg/g for single Cu^2+^, binary Cu^2+^, single Pb^2+^, and binary Pb^2+^ systems, respectively. The adsorption of Cu^2+^ and Pb^2+^ decreased in the binary system; however, the adsorption uptake of Pb^2+^ in the binary system is higher than Cu^2+^. This implies that the presence of Cu^2+^ does not influence the adsorption of Pb^2+^. The adsorption uptake of Cu^2+^ and Pb^2+^ using biochar is compared with other bio-sorbents as presented in Table 4.

## 3. Materials and Methods

### 3.1. Preparation and Characterization of Biochar

Orange peels were collected from a local market in Durban, South Africa. The collected peels were washed thoroughly to remove specks of dust. Thereafter, the peels were dried at 105 °C in an oven for 24 h. The dried biomass was ground and then sieved into particle sizes. The particle size of 150 microns and below was converted into biochar by pyrolysis in a muffle furnace. A particular amount of powdered biomass was put in a crucible and placed in a muffle furnace. The pyrolysis temperature was set at 500 °C using a ramping rate of 15 min and a residence time of 3 h. After the pyrolysis process was completed, the samples were stored in an airtight container for use. 

The physical, chemical, and engineering properties of the prepared biochar were studied. The point of zero charge of the biochar was determined by following the procedure highlighted by the authors of [25]. The biochar was also characterized to determine the functional groups present using an Fourier transform infrared spectroscope (FTIR) (Perkin Elmer, Frontier, Waltham, MA, USA) and scanning electron microscope (SEM) (FEI Nova NanoSEM 230, Lausanne, Switzerland), supported by an energy dispersive x-ray spectroscopy (EDS) anaylsis (Oxford X-Max detector, Oxford, UK and INCA software v7.3, Stuttgart, Germany), which was used to determine the surface structure and elemental composition of the biochar, while an x-ray diffraction spectrophotometer (XRD) analysis was performed to determine the minerals present in the biochar.

### 3.2. Preparation of Synthetic Solution

A synthetic solution of Cu^2+^ and Pb^2+^ ions (1000 mg/L) was prepared in a volumetric flask by dissolving the calculated amount of Cu(NO_3_)_2_·5H_2_O and PbNO_3_ in distilled water. The serial dilution of the stock solution with distilled water was performed to obtain concentrations of the mixed Cu^2+^ and Pb^2+^ ions in the range of 10–200 mg/L. The pH value of the solution was adjusted in the range of 2 to 6 using 0.1 M NaOH and 0.1 M H_2_SO_4_ solutions, and the value was measured with a digital pH meter. All chemicals used were of analytical grade, purchased from Laboratory Analytical Supplies Limited, South Africa.

### 3.3. Batch Adsorption Studies

The effect of the pH value on the removal efficiencies of Cu^2+^ and Pb^2+^ ions from an aqueous solution using biochar was investigated. The effect of the pH value was studied in the pH range of 2 to 6 to obtain the optimum pH value for the experiment. All the experiments were performed in a 250 mL conical flask containing 100 mL of the solution of Cu^2+^ and Pb^2+^ with an adsorbent dosage of 0.1 g at the agitation speed of 120 rpm. At the end of the adsorption process, the supernatant portion was filtered using Whatman filter paper and syringe filters. The samples were then analyzed for Cu^2+^ and Pb^2+^ ions using a micro-plasma atomic emission spectrophotometer (MP-AES, MY 18379001, Agilent, Santa Clara, CA, USA). The percentage of the removal of Cu^2+^ and Pb^2+^ ions at equilibrium and the quantity of ions adsorbed (mg/g) were calculated using Equations (1) and (2), respectively.

(1) $$\%\;Removal=\frac{\left(C_{0}-C_{e}\right)}{C_{0}}\;*\;100$$

(2) $$q_{t}=\frac{\left(C_{0}-C_{e}\right)}{m}V$$

where $C_{0}$ and $C_{e}$ are the initial and final concentration (mg/L), respectively, m is the mass of the bio-sorbent (g), and V is the volume of the solution (L). All the experiments were conducted in triplicates while the average values were reported.

### 3.4. Adsorption Isotherm Studies

The Langmuir and Freundlich isotherm models were used in this study and experiments were conducted with the optimum pH conditions obtained from the sorption of Cu^2+^ and Pb^2+^ ions. The linear form of the Langmuir isotherm model used in this study is represented by the equation below.

(3) $$\frac{C_{e}}{q_{e}}=\frac{1}{q_{max}}C_{e}+\frac{1}{bq_{max}}$$

where *q_max_* is the maximum adsorption capacity of the metal ion (mg/g), $C_{e}$ is the concentration at equilibrium (mg/L), *b* (L/mg) is the Langmuir isotherm constant which shows the affinity of the metal ions for the active sites, while *q_max_* and *b* are obtained from the plot of $C_{e}$/$q_{e}$ against $C_{e}$.

The separation factor *R_L_* is a dimensionless constant obtained from the Langmuir isotherm model to determine whether the sorption system is favorable or unfavorable. The expression for *R_L_* is given as follows:

(4) $$R_{L}=\frac{1}{1+bC_{e}}$$

The separation factor $R_{L}$ can be described as follows: 

$R_{L}$ > 1; unfavorable, $R_{L}$ = 1; Linear, 0 < $R_{L}$ < 1; favorable and $R_{L}$ = 0; irreversible.

The linearized form of the Freundlich isotherm model is expressed as follows:

(5) $$logq_{e}=logK+\frac{1}{n}logC_{e}\;\;$$

where, $q_{e}$ is the adsorbed metal ion quantity per unit weight of the adsorbent (mg/g), $K_{F}$ and *n* are the Freundlich empirical constants (L/g) and $C_{e}$ is the concentration at equilibrium.

### 3.5. Adsorption Kinetic Studies

The adsorption kinetic study explains the adsorption reaction time at the solid–solution interface. The two most popular adsorption kinetic models, pseudo-first-order and pseudo-second-order models, are used in this study. The pseudo-first-order model expression is given below:

(6) $$\log\left(q_{e}-q_{t}\right)=\log q_{e}-\frac{K_{1}}{2.303}t\;$$

The pseudo-second-order model is expressed as follows:

(7) $$\frac{t}{q_{t}}=\frac{1}{K_{2}q_{e}^{2}}+\frac{t}{q_{e}}$$

where $q_{e}$ and $q_{t}$ (mg/g) are the quantity of metal ions adsorbed at equilibrium and at any time *t*. $K_{1}$ and $K_{2}$ are the pseudo-first-order (min^−1^) and pseudo-second-order (g mg^−1^ min^−1^) rate constants, respectively, and t is the contact time (min).

## 4. Conclusions

This study was carried out using orange peel biochar for the removal of Cu^2+^ and Pb^2+^ ions from wastewater in single and binary systems to investigate the effect of the co-existence of these metal ions on the bio-sorption process. The point of zero charge of orange peels before valorization showed that the surface is acidic; however, the orange peel biochar surface charge was 10.03, which is basic. This suggests that the pyrolysis process reduced the acidity of the bio-sorbent due to the heating temperature, resulting in the detachment of alkali metals such as Ca and Mg. In addition, the EDS analysis of the orange peel biochar showed that the bio-sorbent contains a significant amount of carbon and oxygen, indicating that the biomass is incompletely decomposed during the pyrolysis process. Hence, the high content of carbon and oxygen revealed more available active sites on the surface of the bio-sorbent. Furthermore, the results of the FTIR analysis of the orange peel biochar showed significantly pronounced sharp peaks at 1316.4 cm^−1^ and 1015.77 cm^−1^, corresponding to an O-H and C-H stretching vibration. These peaks also imply an increased amount of carbon and oxygen on the surface of the bio-sorbent, thus making it suitable for metal adsorption.

The adsorption uptake of Pb^2+^ was higher than Cu^2+^ in the single and binary systems. This is due to the smaller ionic radius of Pb^2+^, which makes it easier for the metal ion to be adsorbed on the active site. The tendency of Pb^2+^ to have better binding and affinity is therefore inferred to be caused by an increased covalent binding reaction and an ionic radius with a smaller hydrated radius. Hence, the adsorption of Pb^2+^ was not affected by the co-existence of Cu^2+^ in the solution. The adsorption equilibrium experimental data were well described by the Langmuir isotherm model for both single and binary systems, suggesting that adsorption occurred on a monolayer. The kinetic data followed the pseudo-second-order model, which implies that the adsorption mechanism is chemisorption. The adsorption rate was rapid in both single and binary systems, which implies that a smaller column is needed for typical industrial treatment plant applications.

## Figures and Tables

**Table 1:** Linearized isotherm model parameters and their coefficients.

Ion	System	Langmuir			Freundlich		
		b (L/mg)	q_m_ (mg/g)	R^2^	K_f_	n	R^2^
Cu^2+^	Single	2.27	28.06	0.945	1.30	0.31	0.931
Cu^2+^	Binary	1.84	26.83	0.976	1.36	0.31	0.855
Pb^2+^	Single	3.85	30.12	0.980	1.99	0.27	0.962
Pb^2+^	Binary	1.97	27.71	0.933	1.49	0.39	0.818

**Table 2:** Kinetic model parameters obtained from pseudo-first-order and pseudo-second-order models.

	Single			Binary		
Pseudo-First Order	$q_{e}$ mg/g	$K_{1}$ min^−1^	R^2^	$q_{e}$ mg/g	$K_{1}$ min^−1^	R^2^
Metal ion						
Cu^2+^	0.12	12.13	0.696	1.70	0.58	0.626
Pb^2+^	3.34	3.58	0.842	2.47	0.51	0.799
Pseudo-second order	*q_e_*	*K*_2_	R^2^	*q_e_*	*K*_2_	R^2^
	mg/g	g/mg/min		mg/g	g/mg/min	
Metal ion						
Cu^2+^	121.95	0.21	0.950	97.09	0.35	1.000
Pb^2+^	149.25	0.45	1.000	117.65	0.36	1.000

**Table 3:** Characteristics of Pb^2+^ and Cu^2+^ binding strength.

Metal Ions	Atomic Mass	Ionic Radius r	Electronegativity X_m_	Hydration Radius r_hyd_	Hydrolysis Constant (pk_H_)	Covalent Binding X^2^(r_cryst_ + 0.85)
Pb^2+^	207.20	119	2.33	0.401	7.81	7.18
Cu^2+^	63.55	73	1.90	0.419	8.00	6.41

**Table 4:** Comparison of adsorption uptake of Cu^2+^ and Pb^2+^ with other bio-sorbents.

Bio-Sorbents	Cu^2+^ Q_max_ (mg/g)	Pb^2+^ Q_max_ (mg/g)	References
Orange peels biochar	28.06	30.12	This study
Sesame straw biochar	55 *	102 *	[23]
Sugarcane bagasse biochar		86.96 *	[15]
Orange peels biochar		27.86	[15]
Orange peels biochar	65.46 *		[13]
Plum biochar		28.79 *	[24]
Apricot kernel biochar		23.89 *	[24]

* Chemically modified biochar.

## Data Availability

Not applicable.
